# The Effect of Early Treatment with Intravenous Magnesium Sulfate on the Incidence of Cardiac Comorbidities in Hospitalized Stroke Patients

**DOI:** 10.1155/2020/1494506

**Published:** 2020-09-26

**Authors:** Kameron Bechler, Kristina Shkirkova, Jeffrey L. Saver, Sidney Starkman, Scott Hamilton, David S. Liebeskind, Marc Eckstein, Samuel Stratton, Frank Pratt, Robin Conwit, Nerses Sanossian

**Affiliations:** ^1^Keck School of Medicine, USA; ^2^Zilkha Neurogenetic Institute, University of Southern California, USA; ^3^Stroke Center and Department of Neurology, University of California, Los Angeles, USA; ^4^Stanford University, USA; ^5^Department of Neurology, University of Southern California, USA; ^6^Division of Extramural Research, NIH/NINDS, USA

## Abstract

**Background:**

Cardiac adverse events are common among patients presenting with acute stroke and contribute to overall morbidity and mortality. Prophylactic measures for the reduction of cardiac adverse events in hospitalized stroke patients have not been well understood. We sought to investigate the effect of early initiation of high-dose intravenous magnesium sulfate on cardiac adverse events in stroke patients.

**Methods:**

This is a secondary analysis of the prehospital Field Administration of Stroke Therapy-Magnesium (FAST-MAG) randomized phase-3 clinical trial, conducted from 2005-2013. Consecutive patients with suspected acute stroke and a serum magnesium level within 72 hours of enrollment were selected. Twenty grams of magnesium sulfate or placebo was administered in the ambulance starting with a 15-minute loading dose intravenous infusion followed by a 24-hour maintenance infusion in the hospital.

**Results:**

Among 1126 patients included in the analysis of this study, 809 (71.8%) patients had ischemic stroke, 277 (24.6%) had hemorrhagic stroke, and 39 (3.5%) with stroke mimics. The mean age was 69.5 (SD13.4) and 42% were female. 565 (50.2%) received magnesium treatment, and 561 (49.8%) received placebo. 254 (22.6%) patients achieved the target, and 872 (77.4%) did not achieve the target, regardless of their treatment group. Among 1126 patients, 159 (14.1%) had at least one CAE. Treatment with magnesium was not associated with fewer cardiac adverse events. A multivariate binary logistic regression for predictors of CAEs showed a positive association of older age and frequency of CAEs (*R* = 1.04, 95% CI 1.03-1.06, *p* < 0.0001). Measures of early and 90-day outcomes did not differ significantly between the magnesium and placebo groups among patients who had CAEs.

**Conclusion:**

Treatment of acute stroke patients with magnesium did not result in a reduction in the number or severity of cardiac serious adverse events.

## 1. Introduction

Cardiac adverse events are common among patients presenting with acute stroke. Stroke patients, who experience cardiac adverse events (CAEs), have longer hospital stay and increased incidence of morbidity and mortality [1]. Among the most serious cardiac adverse events in hospitalized stroke patients are arrhythmia, acute myocardial infarction, and cardiac arrest [2]. Prophylactic measures for the reduction of cardiac adverse events in hospitalized stroke patients have not been well defined or studied. One possible prophylactic measure is the administration of intravenous magnesium sulfate, a compound with known cardioprotective and neuroprotective properties previously theorized to improve neurological outcomes in acute stroke patients [3].

Hypomagnesemia is associated with an increased incidence of primary and secondary cardiovascular issues including acute myocardial infarction (AMI), atrial fibrillation, arrhythmia following AMI, and cardiac arrest [4–7]. Intravenous magnesium supplementation during the previously stated cardiac events has been shown to offer cardioprotective benefits and decreased morbidity and mortality [4, 8–10]. Furthermore, studies have found that intravenous magnesium can be effective when used prophylactically to stabilize the heart during AMI, coronary artery bypass graft surgery, laryngoscopy, and tracheal intubation [11–13]. These benefits are especially pronounced in individuals with hypomagnesemia [10, 14, 15]. The effect of magnesium sulfate administration and magnesium levels on the incidence of cardiac adverse events in acute stroke patients has not been formally assessed.

We sought to characterize rates and types of cardiac adverse events (CAEs) among acute stroke patients and to investigate the effect of early initiation of high-dose intravenous magnesium sulfate on cardiac adverse events [3]. We sought to determine if there were differences in CAEs among the group treated with acute high-dose magnesium vs. placebo. In a subgroup of subjects who had serum magnesium levels tested, we sought to determine whether achieving target levels of magnesium was associated with a decrease in CAEs. Given the safety profile of intravenous magnesium, a reduction in CAEs in hospitalized stroke patients may have implications for future therapy.

## 2. Methods

We performed a secondary analysis of the prehospital Field Administration of Stroke Therapy-Magnesium (FAST-MAG) randomized clinical trial, a multicenter, phase 3, NIH-NINDS-sponsored, placebo-controlled trial of field initiation of magnesium sulfate for hyperacute stroke. Results of the primary outcomes and detailed methodology have been published previously [3, 16, 17] [18]. The trial was conducted in Los Angeles and Orange counties in California, USA, and included 40 Emergency Medical System agencies, 315 paramedic-staffed ambulances, and 60 acute receiving hospitals. The study protocol was approved by the institute's committee on human research. Off-scene enrolling physician-investigators enrolled patients in the field via cellphone conversation using methods of explicit unformatted consent, consent via legally authorized representative, and exception from informed consent [19] [20].

Magnesium sulfate or placebo was administered in the ambulance starting with a 15-minute loading dose intravenous infusion followed by a 24-hour maintenance infusion started by the Emergency Department nurse in the hospital. This regime rapidly doubled serum magnesium levels and maintaining this increase for the first 24 hours after enrollment. 15 magnesium loading dose consisted of 4.81 g of magnesium sulfate in 60 mL of normal saline, allowing 6 mL for priming and 54 mL (containing 4 g Mg) for administration. The placebo prehospital dose bag consisted of 60 mL of normal saline only. Gravity-controlled tubing with fixed-lumen size was used in the ambulance with the standard height of bag placement at 216 cm, with a rate of infusion controlled at 3.6 mL/min. The maintenance dose consisted of 16 g Mg (or matched placebo). A research pharmacy prepared loading dose infusion bags for paramedic use in the study.

To analyze the effect of early magnesium sulfate administration on CAEs, CAEs were analyzed in a cohort of patients with existing hospital records for serum magnesium blood levels. For these patients, blood magnesium levels were drawn per attending physician orders if their usual practice was to do so in patients with acute stroke or if the patient developed a condition, such as altered mental status, for which they felt serum magnesium evaluation was clinically indicated. These records were obtained by a dedicated group of research staff separately from the original trial data abstraction as magnesium blood levels were not required by the study protocol and to prevent unblinding. In this cohort of patients, additional analysis of CAEs was performed based on the achievement of a target blood magnesium levels in addition to treatment arm group allocation.

Prehospital ambulance services and hospital-receiving sites provided concomitant therapy including supportive care for cardiac conditions per national guidelines and best care practices in addition to the study infusion of magnesium sulfate or placebo.

Binary variables were analyzed using the Pearson chi-square test, and linear variables were analyzed using student *t*-test. Alpha level of 0.05 was used to determine significance. Two-sided *p* values were considered nominally significant. All analyses were considered exploratory and no adjustment for multiplicity was made. A multivariate binary logistic regression model was used to evaluate the association between CAEs and age, blood serum magnesium levels, treatment arm, and diagnosis on the presence of CAEs. Patient clinical and demographic variables (listed in Table 1) were included in the multivariate analysis. Statistical analyses were performed using SPSS version 20.

## 3. Results

Among 1126 patients included in the analysis of this study, 809 (71.8%) patients had ischemic stroke, 277 (24.6%) had hemorrhagic stroke, and 39 (3.5%) with stroke mimics. The mean age was 69.5 (SD13.4) and 42% were female. Median onset to paramedic evaluation time was 16 (IQR 8-35) minutes and 58 (IQR 46-79) minutes for onset to ED arrival. The median prehospital Glasgow Coma Scale (GCS) was 15 (IQR 14-15). Median prehospital LAMS was 4 (IQR 3-5) (Table 1).

Between treatment arms in the trial, 565 (50.2%) received Magnesium treatment and 561 (49.8%) received Placebo. There were no significant differences between the demographic and clinical characteristics of these two groups (Table 1). The mean serum magnesium level for patients in the placebo group was 2.86 (SD 13.4) and for patients in the magnesium group was 3.74 (SD 1.2), *p* < 0.0001 (Table 1). When patients were divided into two groups of achieving or not achieving target serum magnesium level of 3.8 mEg/L, 254 (22.6%) patients achieved the target, and 872 (77.4%) did not achieve the target, regardless of their treatment group. Patients who achieved blood serum magnesium target were significantly older (73.5 vs. 68.3, *p* < 0.0001), more frequently female (52.8 vs. 38.8, *p* < 0.0001), and had more CAEs (18.5 vs. 12.8, *p* < 0.0001) than patients who did not achieve the serum magnesium target (Table 2).

Among 1126 patients, 159 (14.1%) had at least one CAE. The three most common CAEs were new onset atrial fibrillation (4.7%), bradycardia (2.9%), and cardiac arrest (2.2%) (Table 3). Of 159 patients experiencing at least one CAE, 124 (78.0%) had an ischemic stroke, 33 (20.8%) had a hemorrhagic stroke, and 2 (1.3%) were diagnosed with stroke mimic. Patients with CAEs were significantly older than patients without CAEs (75.7 vs. 68.5, *p* < 0.0001), more frequently non-Hispanic (15.7 vs. 25.4, *p* = 0.01), more frequent history of atrial fibrillation (35.8 vs. 20.4, *p* < 0.0001), CAD (28.9 vs. 20.6, *p* = 0.02), MI (17.6 vs. 10.0, *p* = 0.01), valvular heart disease (11.3 vs. 6.8, *p* = 0.04), lower prehospital GCS (*p* < 0.0001), and higher prehospital LAMS (*p* = 0.001) (Table 4).

A multivariate binary logistic regression for predictors of CAEs showed a positive association of older age and frequency of CAEs (*R* = 1.04, 95% CI 1.03-1.06, *p* < 0.0001). When controlled for age and diagnosis, there was no association between treatment group allocation and target serum magnesium status and frequency of CAEs.

**Figure 1:**
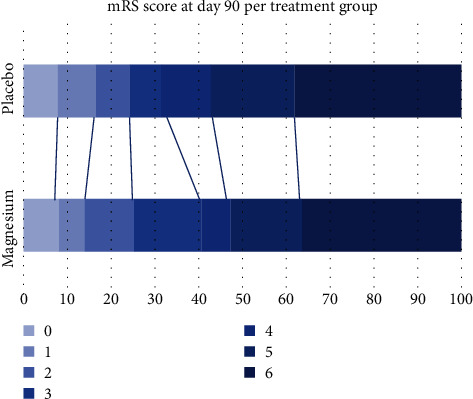
Functional outcomes at 90 days in the magnesium and placebo groups of patients with CAEs, according to score on the Modified Rankin Scale.

**Figure 2:**
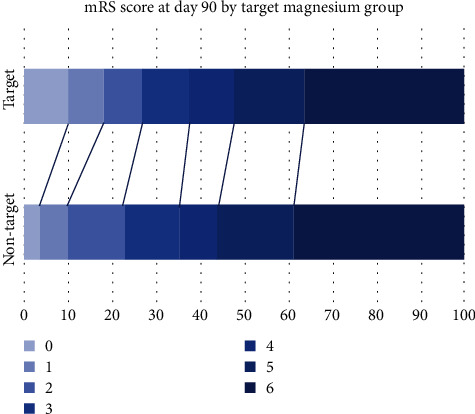
Functional outcomes at 90 days in the target and nontarget groups of patients with CAEs, according to score on the Modified Rankin Scale.

Measures of early and 90-day outcomes did not differ significantly between the magnesium and placebo groups among patients who had CAEs (Table 5). Measures of early outcomes between patients' groups based on target magnesium levels showed a significant difference in GCS recorded by nurse after hospital arrival (13 vs. 15, *p* = 0.04). Measures of 90-day outcomes were not significantly different between patients with serum magnesium levels above or equal to 3.8 mEg/L and below (Table 6). Distribution of 90-day modified Rankin scores for magnesium vs. placebo groups and for magnesium target groups are shown in Figures 1 and 2.

## 4. Discussion

The goal of this study was to characterize CAEs in acute stroke patients and to investigate the effect of early initiation of high-dose intravenous magnesium sulfate on cardiac adverse events in acute stroke patients. Many classic risk factors for cardiovascular diseases were associated with increased rates of CAEs in this population including tobacco use and a history of other cardiovascular diseases like atrial fibrillation, myocardial infarction, coronary artery disease, and valvular heart disease. The most common CAEs in both magnesium and placebo groups were new onset atrial fibrillation, bradyarrhythmia, cardiac arrest, and myocardial infarction. No specific CAE was significantly affected by the administration of magnesium sulfate in this study.

Furthermore, this study did not confirm the primary hypothesis that early initiation of high-dose magnesium sulfate decreases CAEs in stroke patients presenting soon after symptom onset. Neither randomization to the treatment arm nor achieving serum magnesium levels above 3.8 mEg/L impacted the type of CAEs experienced or 90-day outcomes. Patients randomized to the magnesium group and patients with serum magnesium levels above the target were more likely to have at least one CAE. However, a multivariate binary logistic regression controlling for age and diagnosis found no association, positive or negative, between treatment group allocation or target serum magnesium status and the frequency of CAEs.

The findings of this study are congruent with previously published studies showing no overall benefit of magnesium sulfate infusion following myocardial infarction. Although some small studies have asserted that magnesium has cardioprotective benefits, several large studies have found no significance [4]. The MAGIC Trial, which included over 6,000 STEMI patients, found no benefit of early administration of magnesium sulfate on 30-day mortality outcomes [21]. Likewise, the ISIS-4 Trial, including over 58,000 patients, and a more recent trial using data from the Second National Registry of Myocardial Infarction found no benefit of magnesium sulfate administration on mortality outcomes [22, 23].

Among the most potent factors associated with CAEs in this study was age; the mean age of individuals experiencing at least one CAE was over 7 years older than those who had none. This is expected given the higher overall incidence of cardiovascular disease in older individuals compared with younger individuals. Age was also a predictor of attaining target serum magnesium levels above 3.8 mEg/L. This may be explained by a slower renal elimination rate, which is known to the decrease with age. Although creatinine clearance varies between individuals, it has been shown that in older patients above age 40, the rate of renal elimination experiences a steady decline over years [24]. As previously stated, the association between Mg blood levels and the presence of CAEs was rendered insignificant by the multivariate binary logistic regression controlling for age.

In patients who experienced CAEs, there was no significant difference in early (GCS, LAMS, NIHSS) or 90-day (mRS, mortality) outcomes between magnesium and placebo groups. The same is largely true for patients who achieved target serum magnesium levels and those who did not. This finding is expected given the lack of significant association between randomization to the magnesium group or achieving target serum magnesium levels and decreased incidence of CAE in this study. This is consistent with large studies assessing the effect of magnesium sulfate administration on outcomes of patients experiencing acute myocardial infarction [21–23].

There are several limitations to this study that warrant consideration. First, this was not a predefined formal pharmacokinetic study with standardized testing of magnesium blood levels as specified intervals and designated core laboratory. Additionally, while the effect of serum magnesium levels as measured in the hospital were analyzed in this study, the possible confounding effect of serum magnesium levels over the days to months prior to acute stroke was not considered. This may have led to variation in observed magnesium levels due to timing and testing condition variation. However, the opportunistic analysis of magnesium levels drawn during routine care in some but not all patients was previously shown to be the representative of the whole study cohort [18]. Furthermore, it has been shown that commercial magnesium assays show good replicability [25]. Second, the methods employed in this study were originally designed to maximize potential neuroprotective effects of magnesium sulfate administration, not cardioprotective effects, which were analyzed in this study. For this reason, the maximum potential of magnesium sulfate infusion on reducing CAEs in stroke patients may not have been achieved.

In conclusion, 1 in 7 acute stroke patients experienced at least one cardiac adverse event. Early prehospital administration of high-dose magnesium sulfate intravenously did not result in a reduction in the number or severity of cardiac adverse events in acute stroke patients.

## Figures and Tables

**Table 1:** Dichotomized demographic and clinical characteristics of patients in magnesium and placebo groups.

	Overall group (*N* = 1126)	Patients in magnesium group (*n* = 565)	Patients in placebo group (*n* = 561)	*p* value (Mg vs. Pl)
Age (mean, SD)	69.5 (13.4)	69.8 (13.4)	69.2 (13.5)	0.41
Sex female (*N*, percent)	472 (42.0)	243 (43.1)	229 (40.8)	0.44
Diagnosis, *n* (%)				
Acute cerebral ischemia	809 (71.8)	406 (71.9)	403 (71.8)	0.21
Intracranial hemorrhage	277 (24.6)	144 (25.5)	133 (23.7)	
Stroke mimic	39 (3.5)	14 (2.5)	25 (4.5)	
Race, *n* (%)				
White	854 (75.8)	429 (75.9)	425 (75.8)	0.63
Black/African American	157 (13.9)	74 (13.1)	83 (14.8)	
Asian	102 (9.1)	55 (9.7)	47 (8.4)	
Other	12 (1.0)	6 (1.1)	6 (1.1)	
Ethnicity–Hispanic, *n* (%)	270 (24.0)	135 (23.9)	135 (24.1)	0.99
Medical history				
Hypertension, *n* (%)	877 (78.0)	441 (78.2)	436 (77.7)	0.89
Diabetes, *n* (%)	250 (22.2)	138 (24.6)	112 (19.9)	0.62
Hyperlipidemia, *n* (%)	533 (47.4)	274 (48.8)	259 (45.9)	0.34
Atrial fibrillation, *n* (%)	254 (22.6)	125 (22.3)	129 (22.9)	0.83
CAD, *n* (%)	245 (21.8)	127 (22.6)	118 (20.9)	0.52
MI, *n* (%)	125 (11.1)	63 (11.2)	62 (11.0)	0.93
CABG, *n* (%)	30 (2.7)	17 (3.0)	13 (2.3)	0.47
Prior stroke, *n* (%)	86 (7.6)	39 (7.0)	47 (8.3)	0.43
Tobacco use, *n* (%)	199 (17.7)	103 (18.4)	96 (17.0)	0.31
Any alcohol use, *n* (%)	443 (39.4)	225 (40.1)	218 (38.7)	0.63
Time intervals (mins), median (IQR)				
Onset to paramedic evaluation	16 (8-35)	15 (8-32)	17 (8-39)	0.22
Onset to ED arrival	58 (46-79)	57 (46-75)	60 (46-83)	0.10
Severity scores				
Prehospital GCS, `median (IQR)	15 (14-15)	15 (14-15)	15 (14-15)	0.08
Prehospital LAMS, median (IQR)	4 (3-5)	4 (3-5)	4 (3-5)	0.53
Serum magnesium `level mean (SD)	2.86 (1.3)	3.74 (1.2)	1.97 (0.40)	<0.0001
Patients with CAEs, *n* (%)	159 (14.1)	79 (14.0)	80 (14.3)	0.93
Number of CAEs, *n* (%)				
0	967 (85.9)	486 (86.0)	481 (85.7)	0.29
1	136 (12.1)	64 (11.3)	72 (12.8)	
2	21 (1.9)	13 (2.3)	8 (1.4)	
3	2 (0.2)	2 (0.4)	0 (0.0)	

**Table 2:** Dichotomized demographic and clinical characteristics of patients who achieved and did not achieve blood serum magnesium study target within 72 hours (3.8 mEg/L).

	Patients with serum Mg ≥3.8 mEg/L (*n* = 254)	Patients with serum Mg <3.8 mEg/L (*n* = 872)	*p* value
Age (mean, SD)	73.5 (13.1)	68.3 (13.3)	<0.0001
Sex female (*N*, percent)	134 (52.8)	338 (38.8)	<0.0001
Diagnosis, *n* (%)			
Acute cerebral ischemia	178 (70.1)	631 (72.4)	0.13
0.13 intracranial hemorrhage	205 (23.5)	72 (28.3)	
Stroke mimic	4 (1.6)	35 (4.0)	
Race, *n* (%)			
White	203 (79.9)	651 (74.7)	0.06
Black/African American	22 (8.7)	135 (15.5)	
Asian	27 (10.6)	75 (8.6)	
Other	2 (0.8)	10 (1.2)	
Ethnicity–Hispanic, *n* (%)	65 (25.6)	205 (23.5)	0.27
Medical history			
Hypertension, *n* (%)	202 (79.5)	675 (77.5)	0.55
Diabetes, *n* (%)	51 (20.1)	199 (22.8)	0.39
Hyperlipidemia, *n* (%)	111 (43.7)	422 (48.5)	0.10
Atrial fibrillation, *n* (%)	63 (24.8)	191 (21.9)	0.35
CAD, *n* (%)	61 (24.0)	184 (21.1)	0.34
MI, *n* (%)	31 (12.2)	94 (10.8)	0.57
CABG, *n* (%)	6 (2.4)	24 (2.8)	0.82
Prior stroke/TIA, *n* (%)	23 (8.3)	65 (7.5)	0.69
Tobacco use, *n* (%)	41 (16.1)	158 (18.1)	0.51
Any alcohol use, *n* (%)	87 (34.4)	356 (40.9)	0.06
Time intervals (mins), median (IQR)			
Onset to paramedic evaluation	16 (8-32)	16 (8-36)	0.73
Onset to ED arrival	58 (46-77)	58 (46-80)	0.82
Severity scores			
Prehospital GCS, median (IQR)	15 (14-15)	15 (14-15)	0.90
Prehospital LAMS, median (IQR)	4 (3-5)	4 (3-5)	0.48
Serum magnesium level mean (SD)	4.7 (0.9)	2.3 (0.7)	<0.0001
Patients with CAEs, *n* (%)	47 (18.5)	112 (12.8)	0.03
Number of CAEs, *n* (%)			
0	207 (81.5)	760 (87.2)	0.09
1	40 (15.7)	96 (11.0)	
2	7 (2.8)	14 (1.6)	
3	0 (0.0)	2 (0.2)	

**Table 3:** Frequency of most common CAEs (including up to 3 CAEs per patient).

CAEs	Total
Any CAE	218 (12.8%)
New onset atrial fibrillation	81 (4.7)
Bradycardia	49 (2.9)
Cardiac arrest	38 (2.2)
Myocardial infarction	24 (1.4)
Ventricular tachycardia	7 (0.5)
Angina	7 (0.4)
Cardiopulmonary arrest	8 (0.5)
Syncope	3 (0.2)

**Table 4:** Dichotomized demographic and clinical characteristics in stroke patients with and without CAEs.

	Patients with CAEs (*n* = 159)	Patients without CAEs (*n* = 967)	*p* value
Age (mean, SD)	75.7 (11.1)	68.5 (13.5)	<0.0001
Sex female (*N*, percent)	72 (45.7)	400 (41.4)	0.38
Diagnosis, *n* (%)			
Acute cerebral ischemia	124 (78.0)	685 (70.8)	0.18
Intracranial hemorrhage	33 (20.8)	244 (25.2)	
Stroke mimic	2 (1.3)	37 (3.8)	
Race, *n* (%)			
White	119 (74.8)	735 (76.0)	0.97
Black/African American	21 (13.2)	136 (14.1)	
Asian	17 (10.7)	85 (8.8)	
Other	2 (1.2)	10 (1.0)	
Ethnicity–Hispanic, *n* (%)	25 (15.7)	245 (25.4)	0.01
Medical history			
Hypertension, *n* (%)	131 (82.4)	746 (77.2)	0.18
Diabetes, *n* (%)	44 (27.7)	206 (21.3)	0.08
Hyperlipidemia, *n* (%)	86 (54.1)	447 (46.3)	0.07
Atrial fibrillation, *n* (%)	57 (35.8)	197 (20.4)	<0.0001
CAD, *n* (%)	46 (28.9)	199 (20.6)	0.02
MI, *n* (%)	28 (17.6)	97 (10.0)	0.01
CABG, *n* (%)	3 (1.9)	27 (2.8)	0.78
Valvular heart disease, *n* (%)	18 (11.3)	66 (6.8)	0.05
Prior stroke, *n* (%)	12 (7.5)	74 (7.7)	1.0
Tobacco use, *n* (%)	19 (11.9)	180 (18.6)	0.04
Any alcohol use, *n* (%)	56 (35.2)	579 (40.1)	0.25
Time intervals (mins), median (IQR)			
Onset to paramedic evaluation	16 (7-36)	16 (8-35)	0.98
Onset to ED arrival	58 (46-85)	58 (46-78)	0.88
Severity scores			
Prehospital GCS, median (IQR)	15 (12-15)	15 (14-15)	<0.0001
Prehospital LAMS, median (IQR)	5 (3-5)	4 (3-5)	0.001
Serum magnesium level mean (SD)	3.0 (1.4)	2.8 (1.2)	0.05

**Table 5:** Outcomes for patients with CAEs in magnesium and placebo groups.

	Total (*n* = 159)	Patients with CAEs—magnesium group (*n* = 79)	Patients with CAE—placebo group (*n* = 80)	*p* value
Early outcomes				
Nurse GCS, median (IQR)	14 (11-15)	15 (11-15)	14 (11-15)	0.55
Nurse LAMS, median (IQR)	5 (3-5)	5 (3-5)	5 (2-5)	0.42
Nurse NIHSS, mean (SD)	15.1 (9.2)	15.9 (9.8)	14.3 (8.6)	0.27
90-day outcomes				
mRS 90 d 0-1, *n* (%)	23 (14.6)	10 (12.7)	13 (16.5)	0.65
mRS 90 d 0-2, *n* (%)	39 (24.7)	20 (25.3)	19 (24.1)	1.0
mRS 90 d, mean (SD)	4.1 (2.0)	4.0 (2.0)	4.2 (2.0)	0.58
Mortality 90 d, *n* (%)	59 (37.1)	28 (35.4)	31 (38.8)	0.73

**Table 6:** Outcomes for patients with CAEs in patients by serum magnesium target group.

	Patients with CAEs—with serum Mg ≥ 3.8 mEg/L (*n* = 47)	Patients with CAE—with serum Mg < 3.8 mEg/L (*n* = 112)	*p* value
Early outcomes			
Nurse GCS, median (IQR)	13 (10-15)	15 (12-15)	0.04
Nurse LAMS, median (IQR)	5 (3-5)	5 (3-5)	0.68
Nurse NIHSS, mean (SD)	17.1 (10.4)	14.3 (8.6)	0.08
90-day outcomes			
mRS 90 d 0-1, *n* (%)	3 (6.4)	20 (18.0)	0.08
mRS 90 d 0-2, *n* (%)	30 (27.0)	9 (19.1)	0.32
mRS 90 d, mean (SD)	4.4 (1.8)	4.0 (2.1)	0.26
Mortality 90 d, *n* (%)	18 (38.3)	41 (36.6)	0.85

## Data Availability

The main FAST-MAG trial database and materials have been made publicly available at the NIH-NINDS Archived Clinical Research Datasets and can be accessed at https://http://www.ninds.nih.gov/Current-Research/Research-Funded-NINDS/Clinical-Research/Archived-Clinical-Research-Datasets.
